# Excess gestational weight gain: an exploration of midwives’ views and practice

**DOI:** 10.1186/1471-2393-12-102

**Published:** 2012-09-27

**Authors:** Jane C Willcox, Karen J Campbell, Paige van der Pligt, Elizabeth Hoban, Deborah Pidd, Shelley Wilkinson

**Affiliations:** 1 Centre for Physical Activity and Nutrition Research, School of Exercise and Nutrition Sciences, Deakin University, 221 Burwood Hwy, Burwood, Victoria, 3125, Australia; 2 School of Health and Social Development, Deakin University, Melbourne, Australia; 3 Mercy Hospital for Women, Melbourne, Australia; 4 Mater Mothers’ Hospital, Brisbane, Australia; 5 Mater Medical Research Institute, Brisbane, Australia

**Keywords:** Gestational weight gain, Pregnancy, Midwives, Weight, Qualitative research

## Abstract

**Background:**

Excess gestational weight gain (GWG) can affect the immediate and long term health outcomes of mother and infant. Understanding health providers’ views, attitudes and practices around GWG is crucial to assist in the development of practical, time efficient and cost effective ways of supporting health providers to promote healthy GWGs. This study aimed to explore midwives’ views, attitudes and approaches to the assessment, management and promotion of healthy GWG and to investigate their views on optimal interventions.

**Methods:**

Midwives working in antenatal care were recruited from one rural and one urban Australian maternity hospital employing purposive sampling strategies to assess a range of practice areas. Face-to-face interviews were conducted with 15 experienced midwives using an interview guide and all interviews were digitally recorded, transcribed verbatim and analysed thematically.

**Results:**

Midwives interviewed exhibited a range of views, attitudes and practices related to GWG. Three dominant themes emerged. Overall GWG was given low priority for midwives working in the antenatal care service in both hospitals. In addition, the midwives were deeply concerned for the physical and psychological health of pregnant women and worried about perceived negative impacts of discussion about weight and related interventions with women. Finally, the midwives saw themselves as central in providing lifestyle behaviour education to pregnant women and identified opportunities for support to promote healthy GWG.

**Conclusions:**

The findings indicate that planning and implementation of healthy GWG interventions are likely to be challenging because the factors impacting on midwives’ engagement in the GWG arena are varied and complex. This study provides insights for guideline and intervention development for the promotion of healthy GWG.

## Background

Over one third to a half of women enter pregnancy overweight or obese, with a Body Mass Index (BMI) ≥25 kg/m^2^[1-4]. This is compounded by 30-50% of women in developed countries, across all BMIs, exceeding recommended guidelines for gestational weight gain, (GWG) with the prevalence significantly increasing in the last two decades [5-8]. Many studies suggest that excess GWG, across all pre-pregnancy BMIs, is associated with short and long term negative health outcomes for maternal and child health [9,10] and highlight the need for prevention. Excess GWG increases the likelihood of antenatal hypertensive disorders, gestational diabetes, atypical delivery outcomes and failure to breastfeed [9,11-15], and is associated with increased neonatal mortality, [16] and neonatal, infant and later life adiposity [11,13]. Further, increased and persistent postpartum overweight, higher weight in subsequent pregnancies and increased risk of overweight and obesity in later adult life are all associated with excess GWG, with each being a known risk for the development of cardiovascular disease and type II diabetes [5,9]. Currently there are no Australian GWG guidelines and the revised 2009 Institute of Medicine (IOM) guidelines [17] are most commonly adopted in developed countries in the absence of country specific guidelines [17,18].

Excess GWG is determined by a myriad of factors, including pre-pregnancy BMI, nutrition, physical activity, healthy pregnancy guideline awareness and psychosocial factors [17]. These predictors are potentially modifiable and opportunities exist to provide interventions to promote healthier GWG. Effective interventions may positively influence maternal and infant health outcomes, altering the future weight trajectory for two people and potentially impacting the intergenerational obesity cycle [19]. Evidence suggests that an antenatal intervention is opportune [20] and women are likely to be particularly receptive to advice during pregnancy [21]. However, resources in hospitals and in the community aiming to promote healthy GWG are limited [22]. A number of systematic reviews of interventions promoting healthy GWG have reported weak or inconclusive evidence regarding effectiveness [23-26], however recent meta-analyses report that interventions based on physical activity and diet counselling, combined with weight monitoring, appeared to be successful in reducing excess GWG [27,28].

While the antenatal and delivery care for women will differ across countries and systems, midwives tend to be one of the key service providers, along with physicians and obstetricians [20,29]. Opportunities exist for midwives to support women to achieve positive lifestyle changes that may promote healthy GWGs [18,20,30]. Understanding midwives’ views, attitudes and practices around GWG will provide insights to inform the development of practical, time efficient and cost effective ways of supporting health providers, including for midwives, to promote healthy GWGs with the potential to maximize best outcomes for pregnant women.

The research regarding midwives’ views and practices concerning GWG is limited. A small number of studies have examined midwives and other health providers views, and approaches to antenatal weight measurement [18,31], services for obese women [32,33] or views of GWG management [20,31,34,35]. These studies report a broad range of views on weighing women, the importance of excess GWG and responses to excess GWG. Further, health providers were unsure of what to advise women regarding appropriate GWG and described barriers to engagement including insufficient training, concern about the sensitivity around weight, and the perception that counselling is ineffective. In many of the studies from the Australia [31], USA [20,34] and UK [32-34] midwives were a subset of these samples and only two UK studies have primarily sampled midwives [18,35]. In addition, the USA has GWG guidelines in place, whereas in Australia there are no GWG guidelines so it is important to assess midwives’ views independently.

The aims of this study were to explore midwives’: approaches to the assessment and management of healthy GWG; views on their role in identifying, managing and promoting healthy GWG and associated lifestyle factors and; views on optimal interventions to facilitate healthy GWG.

## Methods

### Study design

Qualitative descriptive research methodology [36,37] utilising face-to-face semi-structured interviews, using an interview guide were employed to obtain in-depth data from consenting midwives.

### Study participants

The study recruited midwives who worked in, or managed, antenatal clinics in two maternity hospitals in Victoria, Australia, one rural and one urban. Purposive sampling [38] was used to ensure coverage of a broad range of practice areas. The sample of midwives sought included one Antenatal Clinic Director from each site and midwives across both hospitals. The sample size was informed by a similar study with general practitioners (GPs) that found data saturation occurred at 10–12 participants [39]. Midwives were invited to participate in interviews via written or face-to-face invitation after being informed of the study by their department managers.

### Data collection

Face-to-face interviews with midwives were conducted by JW using a standardized interview guide (see Additional file 1). The content of the interview guides was informed by an analysis of the literature and a previous study with GPs [39]. The Antenatal Clinic Director’s interviews aimed to provide an overview of the hospital context in relation to GWG that included policies, clinic guidelines and programs along with a personal perspective on GWG. Policies and guideline documents were examined during the interview with the Director in order to contextualize midwifery practice and provide an independent level of data for data triangulation [40] against the data collected from the midwives. Semi-structured and structured questions to elicit midwives’ views, attitudes and practices around GWG, as well as their thoughts on optimal interventions, were investigated during the interviews. Common themes explored included: focus of the first antenatal clinic visit; practice and advice regarding GWG and weighing; midwives perceived roles in lifestyle education, including GWG; and how midwives could be best supported to provide healthy lifestyle advice and support to pregnant women. In addition, socio-demographic characteristics of all participants were collected, which included the midwife’s role in, and length of employment at the health facility. The interviews were digitally recorded, with the consent of the participants, and transcribed verbatim. Ethics approval was obtained from both hospital sites and Deakin University Human Research Ethics Committee.

### Data analysis

Data immersion, coding, category creation and thematic analysis were used to find repeated patterns of meaning across all data sets [41-44]. The researchers used an inductive approach using raw data to derive themes through interpretations made from the raw data [45]. Inter-rater reliability [46] was confirmed by two researchers carrying out the data analysis to reduce researcher bias during the thematic development phase. The final category system produced was agreed to by both researchers and accepted as being representative of the data.

## Results

### Study participant characteristics

Fifteen female midwives participated in the study. Three of the four possible midwives from the rural hospital and 11 of 25 midwives from the urban hospital consented and were interviewed. One additional midwife from the rural setting consented to be interviewed but withdrew due to illness. The Antenatal Clinic Director of the urban hospital (herself a midwife) was interviewed however, in the rural setting the midwives shared the administrative responsibility, and thus the administration related questions were shared among the participant midwives. Saturation of themes was evident after nine interviews, however the remaining interviews were carried out to ensure all practice areas were included and to confirm data saturation.

The midwives worked across a diverse range of antenatal practice areas including: hospital antenatal clinics (n = 5); community outreach clinics (n = 3); midwifery continuity clinics (n = 3); shared care (joint GP and antenatal clinic) (n = 1); perinatal clinic (n = 1); family birthing unit (n = 1) and Director of Antenatal Clinic (n = 1). The participants’ experience working as a midwife averaged 21 years (range 3 – 37 years). The interview length ranged from 30 minutes to 75 minutes.

### Emergent themes

The thematic content analysis identified a number of overarching themes and subthemes. Three key themes emerged: 1. GWG being a low priority; 2. midwives concern for the physical and psychological welfare of women and; 3. the central role for midwives in the education process with opportunities for additional support to promote healthy GWG. The Antenatal Clinic Director quotes have not been differentiated from the Midwife quotes due to the possibility of interviewee identification and thus breach of anonymity.

#### Theme 1: Gestational weight gain is a low priority for midwives

With the many competing interests in antenatal clinics, GWG was perceived by many midwives to be of low priority. A range of factors contributed to this perception, ranging from absence of policies through midwife beliefs regarding GWG, and their support to engage effectively on this topic. Contributing factors included: practices, policies and views limiting the weighing of women and provision of GWG guidelines; perceptions regarding pregnant women’s low levels of interest in weight; limited education of midwives regarding GWG; time limitations for education of pregnant women; and perceptions of limited allied health services, such as Dietetic and Physiotherapy resources.

*a. Low incidence ofweight monitoring*

In both hospitals midwives often weighed women at the first antenatal visit, and sometimes BMI was calculated, primarily as a risk stratification strategy. Generally, a woman’s weight was not re- measured during her pregnancy unless the woman was defined as “high risk” (BMI > 35 kg/m^2^ or presenting with a co-morbidity) at the outset. The urban hospital had a formal weighing policy [47] and GWG guidelines available to staff on the internal intranet. This hospital’s policy and practice at the hospital discouraged weighing women after the first antenatal visit and this was reflected in some midwives’ views. Further, the policy encouraged the provision of the IOM GWG guidelines based on BMI [17]. Despite the presence of weighing and GWG guidelines policy in the urban hospital and the absence in the rural hospital, there did not appear to be a significant difference in views and practices between both midwife groups. Both groups exhibited diverse views and practices.

"“ (I don’t thinkweighing is) relevant; we’rejust going by clinicalindications.” (Urban midwife 2)"

When midwives were asked about routine weighing practices, two-thirds said they did not consider that routine weighing of pregnant woman was important. The midwives stated there was “no evidence” to support routine weighing and that measurements did not provide useful clinical information. In addition, midwives reported feeling that routine weighing may cause women psychological distress. The acknowledgement of change in pregnant women’s weight was seen to come primarily from the women or midwives’ observation.

"‘… the research supportsthat they don’t reallyneed to be weighedat every appointment. Itdoesn’t really gain muchinformation out of it.” (Urbanmidwife 9)"

"“Too much stigma associatedwith it…It’s embarrassing forthe patient; they seeit as a kindof test, how goodthey’ve been or howbad they’ve been.” (Urban midwife7)"

"“…here we can providecontinuity of care, soI can actually seethe same women forall of her appointmentsapart from one ortwo because she’s seeingher doctor. So Ican actually gauge them(visually), how much they’vebeen putting on.” (Urbanmidwife 9)"

However, the remaining third of midwives supported weighing during pregnancy and felt that weighing women at each antenatal visit allowed them to track GWG, particularly in high risk women such as those of high and low BMIs or those at risk of weight loss. Recording routine weights on women’s care plan was seen as a practice that would normalise weighing and help trigger conversation with women regarding weight and lifestyle behaviours.

"“We were told thatby weighing women, itdoesn’t tell us aboutgood foetal outcomes, sowe stopped…. But weforgot about the processfor women, and whatare the outcomes forwomen if we doweigh them and knowwhat weight they areat the end ofthe pregnancy.” (Urban midwife 6)"

"“(weighing)… instigates a conversationsometimes at each visit,whereas here women don’tget weighed as ageneral rule. …so youdon’t have that conversation,or you don’t havethat prompting.” (Urban midwife 5)"

The challenges associated with the identification of abnormal weight changes were raised by a few urban midwives. They acknowledged that weight changes cannot be identified when women are not weighed routinely.

"“…they’re not identified. Wewould have no ideawhat people put onin pregnancy.” (Urban midwife 5)"

*b. Diverse views regardingprovision of pregnancy GWGguidelines*

Midwives expressed mixed feelings regarding whether GWG guidelines should be provided to women. Two-thirds of the midwives indicated they did not consider it necessary to provide pregnant women with GWG guidelines unless the woman asked for them. Midwives’ reluctance to discuss weight reflected a perceived lack of evidence regarding GWG, weight not being a priority for the midwives and concerns that women may become fixated on their weight during pregnancy. Consistent with the views regarding routine weighing, high risk women with high BMI and concurrent diseases such as diabetes were seen to be the exception.

"“….I think the trendis not worry somuch how much weightgain you have rightthrough unless there’s othermedical issues involved suchas hypertension and smokingand all that sideissues.” (Rural midwife 2)"

"“I guess it’s thatthing where you knowthe woman is goingto put on weightand they do eatmore so generally Iguess I wouldn’t feelthat I would needto.” (Rural midwife 3)"

"“(Providing guidelines). should bealways research based, butI don’t think itis.” (Urban midwife 6)"

The third of midwives who provided GWG guidelines to women cited foetal and maternal outcomes as their main reason for doing so, along with the habit of providing weight guidelines to women.

"“I feel that theyall should be givenso that they havea rough idea ofwhat is normal andnot normal, so they’rehaving a proper dietand exercise.” (Urban midwife2)"

"“So I think we…… need some guidelines” (Rural midwife 2)"

The GWG guidelines provided to women during antenatal care by all midwives, either voluntarily or if asked, varied greatly. A few urban midwives provided women with individual guidelines related to pre-pregnancy BMI, such as the IOM guidelines [17], but the majority provided highly varied ranges for example 10-20 kg or 10–14 kg. Not providing GWG information to women was in contrast to the policy supporting the provision of GWG guidelines at the urban hospital [47].

Half the midwives said that women sought weight gain advice during antenatal care and half noting that weight was rarely raised in consultations. Some midwives felt that healthy weight women were more likely to ask about GWG guidelines. A few midwives shared their personal strategies on discussing GWG and normalising the healthy GWG. The most common strategies involved focusing on the benefits of healthy GWG for the foetus and differentiating the pregnancy weight gain from weight gained through a positive energy balance.

"“I tell them thatgaining weight in pregnancyis completely different togaining weight when youeat too much cake.” (Urbanmidwife 7)"

*c. Excess GWG notseen to be commonor problematic by many*

Most midwives considered excess GWG to be uncommon with the exception of women deemed at “high risk”. In addition, many communicated that they did not see excessive GWG as a significant health issue for women. However, it was also highlighted by some that GWG was impossible to detect since weighing pregnant women was uncommon.

"“(Excess GWG) is unusualfrom my experience…” (Urban midwife1)"

"“…your baby’s an appropriatesize then no-one’s goingto be too concernedif there’s a 20kilo weight gain.” (Urban midwife5)"

"“…they’re not identified. Wewould have no ideawhat people put onin pregnancy.” (Urban midwife5)"

In contrast, the midwives who considered excessive GWG problematic were concerned about maternal and foetal outcomes. There was a sense that the emphasis on GWG had been inappropriately played down over recent years. In addition, concern was expressed that excess GWG compounded associated problems for those already overweight or obese.

"“But I think wesort of ignore thefact that a lotof the girls havestarted heavier. We area fatter population sowe still have theproblem of really bigwomen being pregnant, andgetting to the endof the pregnancy theyhave other problems aswell they get toobig.” (Rural midwife 2)"

When midwives were prompted to identify important implications of excess GWG, the most common responses related to gestational diabetes, preeclampsia, inability to palpate the foetus and complicated deliveries. Two midwives mentioned foetal health implications, including macrosomia.

*d. Limited resources toaddress GWG and lifestylebehaviours*

The midwives identified a lack of time and resources, such as dietetic services, as key limitations enabling them to address healthy GWG and lifestyle issues with the women. Midwives are required to address a large number of issues during antenatal consultations including assessment of medical, family, pregnancy and psychological history as well as provision of pregnancy information, antenatal tests, procedures and bookings. Midwives considered they had limited time available for discussions about GWG and healthy lifestyle. The late timing of the first antenatal visits (often occurring after the first trimester) was sometimes seen to preclude education when it would have been most appropriate. In addition, a reluctance to bombard women with excess information influenced midwives’ decisions about what topics to discuss during visits.

"“. when you’re ona time efficiency….you can’treally think of everytopic, because every topicin pregnancy has becomethe most important, becausethere’s always a smokingprocess going on. There’sthe alcohol intervention process,so everything becomes themost important thing inpregnancy.” (Urban midwife 5)"

"“.they are blown awayby how much wegive them in theearly visits.” (Urban midwife 1)"

As noted, the limited resources for dietetic and physiotherapy services were seen to constrain interventions for healthy GWG and lifestyle issues, reducing the ability for antenatal services to intervene even if a need was identified.

"“.our Dietetics have anappointment system .(and). thoseappointments are hard toget because they takea long time andby the time youget there you couldbe half way throughthe pregnancy. ” (Urbanmidwife 10)"

One midwife felt that the limited dietetic and physiotherapy resources available to them in the public health system has resulted in a redefinition of “at risk” or “healthy” pregnancy weight because only those women with BMIs > 35/kg/m^2^ were chosen for interventions and education. Therefore women with BMIs 25 kg/m^2^ to 35 kg/m^2^ were redefined as “normal”.

"“.there’s a lot ofissues for women around…being fat and weightgain and pregnancy whichwe normalise” (Rural midwife1)"

#### Theme 2 Concern for physical and psychological health of pregnant women

Midwives articulated a concern for the physical and psychological health of pregnant women in general. However, their greatest concern was for possible psychological ramifications of weight related discussions and interventions.

*a. Concern for thepsychological impacts of weightdiscussions and women’s inappropriateviews on weight gain*

It was a common view among the midwives that many women were inappropriately concerned about putting on too much weight during pregnancy. This concern was mirrored in the antenatal weighing policy of one hospital [47]. At this urban hospital the midwives felt that women were controlling their GWG through inappropriate strategies, such as restricted eating, but did not cite evidence to support the supposition. Hence, with a desire to “do no harm” some midwives were concerned about perceived psychological ramifications if weight and GWG were discussed and monitored at routine antenatal visits. Further, there was concern that women would become anxious about their weight, or actively lose weight which would have adverse effects on the mother and foetus. This was expressed as the prime reason for not discussing GWG.

"“I think it stressesa lot of pregnantwomen out. I finda lot of womenare fixated on weightand how much theyshould be gaining.” (Urbanmidwife 3)"

"“Women were getting veryanxious and they weregetting obsessed about (weightgain) and I thinkthat added extra anxiety,they’re already anxious withtheir pregnancy.” (Urban midwife2)"

Other midwives recognised the co-morbidities associated with excessive GWG, such as poor delivery and foetal outcomes, caused by not informing and/or supporting women to achieve these goals and the need for good health outcomes.

"“You know, we canbe nice about itall, but at theend of the day,we want good foetal,good maternal outcomes.” (Urban midwife6)"

*b. Concern for thephysical health of women*

The majority of midwives expressed deep concern about the physical health of their patients. In particular, a few expressed concerns about the increasing incidence in overweight and obesity in the community and their desire for an intervention to reduce women’s weight pre-pregnancy.

"“I consider it (pre-pregnancyoverweight and obesity) areally big (issue), probablyacross my midwifery timeone of the biggestissues that’s out thereat the moment.” (Urban midwife7)"

#### Theme 3 Midwives are central to healthy lifestyle education process and opportunities exist for support to promote healthy GWG

All midwives viewed themselves as part of a team of antenatal colleagues who were responsible for the promotion of healthy lifestyle behaviours, including healthy GWG. When asked about how midwives could be best supported to deliver healthy weight and lifestyle behaviour education, a number of models were suggested.

*a. Key providers oflifestyle behaviour education*

Despite some midwives expressing concerns about healthy GWG and their role in its promotion, the midwives unanimously saw themselves as having responsibility for education and interventions around GWG and lifestyle issues. This was seen to be a responsibility shared with obstetricians, general practitioners and other health providers that pregnant women consulted. The need for consistent messages and education along with multidisciplinary care was also mentioned.

"“So it’s all ourjobs and the ideawould be to worktogether and with ourmost difficult clients usingsupport such as Dieteticsand whatever it isthe woman needs.” (Urbanmidwife 7)"

Most midwives discussed some lifestyle behaviours during pregnancy, however, they considered that Listeria infection and vitamin and mineral intake and supplementation to be the most important. This was followed by advice regarding “general healthy nutrition”, avoidance of alcohol and smoking and the importance of physical activity.

*b. Lack of confidencein addressing weight andGWG*

The majority of midwives thought that conversations with women regarding their weight were difficult, reflecting a negative social construction around weight. It was therefore often easier to avoid raising weight as a concern during antenatal consultations.

"“.weight is a difficultone. It’s easier tobring it up ifyour blood pressure’s high,or you’ve got proteinin you urine. Butwhen you’ve got tosay to someone “You’rea little bit overweightfor midwives to lookafter.” .it’s not anice thing to say,but I think…they understandif you discuss itin a clinical riskmanner.” (Urban midwife 6)"

"“I know myself Iam so euphemistic aboutthe conversation.” (Urban midwife 2)"

Midwives felt that it was important for them to develop the communication skills needed to establish rapport with women that would enable them to have conversations around weight so that discussions were positive, non-judgemental and did not infer blame.

"“ I hear younggrads say all thetime “oh I don’tknow how to talkto women cos theirBMI is high” andI think to myselfhave you never learntabout putting your judgementto one side andgiving facts and lettingpeople see you meanwhat you say, thatyou’re not there judging” (Urbanmidwife 7)"

*c. Support for midwivesto promote healthy GWG*

A model for education and support for midwives to increase their knowledge, skills and opportunity was the most commonly suggested way to help midwives promote and encourage healthy GWGs. Some participants recommended additional education and training for midwives around GWG and others saw greater opportunity for intervention which could occur during longer antenatal consultations.

"“Probably for midwives tohave a lot moreeducation on what weshould be saying towomen and what weshould be doing, becausewe are at theforefront of seeing thesewomen.” (Urban midwife 9)"

The need for longer and individualised antenatal consultations was underpinned by the midwives’ perception that women wanted individual consultations with midwives, continuity of care and relationship and trust building.

Another model to support midwives was the implementation of healthy GWG detection and management policies that would flow down to practice changes, where there was an expectation that GWG would be discussed.

"“I know that’s whatI’d like to see,these triggers that comeup. Because I knowfor the smoking, there’sthe trigger point whereyou must ask thequestions, and it’s partof what you doat every visit.” (Urban midwife6)"

Models targeting women to promote healthy GWG were suggested. These models would ideally utilise multidisciplinary antenatal group sessions employing midwives, dietitians and physiotherapists. Drop in services for ‘high risk’ groups such as refugees and young mothers were suggested by others.

When the midwives were prompted to consider whether some of the new technologies such as the internet, telephone counselling and short message service (SMS) interventions could be used in this context, the midwives favoured the internet and SMS interventions. However, some expressed concern over quality of information and the ability for some women to access the technologies. Others felt that the introduction of these interventions may augment services, increase consistency of information and provide improved access to ‘at risk’ groups.

"“People are hooked intothe internet these days.That’s where they areseeking a lot ofinformation. ” (Urban midwife3)"

"“…doesn’t matter what economicclass people come from,they’ve always got amobile. But if it’scoming to their phone,they’re always going toread a message, whichis a really goodway to get tothese people……….” (Urban midwife9)"

*d. Features and contentof an optimal interventionto promote healthy GWG*

Continuity of care was considered an optimal feature to promote healthy GWG with women seeing the same midwives or health professionals at each visit.

"“I sometimes think it’sbetter to have thatpersonal input from someoneyou’ve actually built upa rapport with.” (Urbanmidwife 1)"

Interventions connected and branded to the antenatal clinic and consistency of messages were seen to be central features in a contiguous approach.

"“……even hospital (nutrition based)internet sites would begood.” (Urban midwife 3)"

Healthy eating, followed by physical activity and the provision of individual GWG parameters, were the topics perceived to be the most important for inclusion in an intervention. Furthermore, supporting women to learn from health providers and other women was seen to be crucial to intervention success.

"“… the food groupeating isn’t enough, itisn’t enough to tellsomeone who doesn’t understandabout nutrition that thisis the way you’resupposed to eat. Havingsomeone sit down toteach them about what’son the back ofpackaging and how toread the packaging andwhat is a goodfood and giving themexamples of what ameal is much morebeneficial” (Urban midwife 10)"

"“Eat from a widefood group. Exercise asa balance in yourlife. And, I’m tryingto think of theright way of puttingit, don’t go toextremes.” (Urban midwife 7)"

## Discussion

In this study, midwives demonstrated a diverse range of views and practices regarding GWG, such as the detection and assessment of GWG, the provision of GWG guidelines and the understanding of the clinical significance of excess GWG. Notwithstanding, midwives universally identified some negative implications of pregnant women having excess GWG. Midwives expressed the desire to spend more time with women during antenatal clinics so they could discuss associated lifestyle behaviours. They suggested clinical interventions if excess GWG was detected and they contributed ideas regarding the ways in which they, and pregnant women, could be further supported to improve lifestyle behaviours.

The midwives’ diverse, and often contradictory, views with low priority given to GWG alongside a concern for women’s physical health and a belief in the role of midwives in the promotion of a healthy lifestyle, are corroborated by related research. In a recent UK online survey of 241 midwives, only 15% of respondents offered personalised GWG advice based on the woman’s diet and physical activity [35]. This was despite 77% believing it was appropriate and 69% believing it was feasible to offer such advice. Interestingly one challenge to providing GWG advice, suggested by this cohort, was the midwives’ personal weight and weight management issues. In an older UK study differences in the perceived relevance of weighing influenced whether midwives would act in response to “abnormal” GWG, and whether they advised women to gain or lose weight during pregnancy [18]. These and other studies [20,34,39] suggest a wide variation in attitudes and practices of health providers, including midwives, regarding GWG detection, education and intervention. This may come from a lack of nationally recognised guidelines for weighing and GWG, limited health professional education and changes in practice resulting in non-evidence based approaches to care and advice.

The short and long-term implications of excess GWG on both mother and child are increasingly evident [9]. Interestingly, all the midwives interviewed acknowledged some short-term maternal complications, however, only two midwives mentioned short-term foetal health implications and one a long-term maternal complication. This finding is consistent with study of Australian GP’s beliefs and practices regarding GWG [39] where GPs indicated a limited range of GWG associated complications. These findings suggest that midwives may not be fully aware of the health impacts of GWG. Further research is required to help understand the best method of increasing health providers’, including midwives’, understanding of the risks and complications associated with excess GWG.

This study emphasised the lifestyle behaviour priorities of the midwives. However, their priorities were at odds with the prevalence and outcome data for lifestyle behaviours during pregnancy. For example, midwives placed greater emphasis on a discussion of the prevention of Listeria contamination and the promotion of multivitamin use over general nutrition advice, physical activity and weight gain advice. These findings resonate with the UK survey of 672 midwives [18] who ranked “normal weight gain” as the least important focus of nutrition advice for pregnant women. This may reflect their understanding of, ease of delivery and comfort with various lifestyle messages. The acute versus chronic nature of sequelae is also likely to be important. A refocus of priorities is required.

Irrespective of healthy GWG, evidence suggests that women are not meeting dietary [3,22,48] or physical activity [3] guidelines. Moreover, it appears that most pregnant women are seeking nutrition information [22]. More than half (55%) of a sample of 411 Australian pregnant women, who were representative of a busy urban maternity hospital population, identified healthy eating as a priority and wanted nutrition information [22]. This illustrates the need for those working with pregnant women to reframe lifestyle behaviour education to reflect a more comprehensive view of health and wellness. Antenatal guidelines and education should be developed in the context of all lifestyle behaviours, not just Listeria prevention or GWG guidelines, to minimise the problem where particular issues are given priority over others. Research is required to develop effective interventions that address nutrition and physical activity while encompassing the specific requirements of pregnancy.

Two dominant barriers to the provision of healthy GWG and lifestyle behaviour advice identified by the midwives were a lack of confidence regarding how to provide weight advice and concern over causing psychological harm. While the concern for fostering anxiety is present in the literature [18,20], the focus on this issue as a cause of active and inappropriate weight reduction remains unclear. These views may be a reflection of increasing awareness of body image and disordered eating behaviours among the population, the hospital policy from where the majority of the sample was drawn mentions that women “*may try to controlweight gain through inappropriatestrategies*” [47], and/or the desire of midwives to do “no harm” in their interactions with women. However, evidence suggests that failure to acknowledge the issue of excess weight is likely to reinforce the problem [32]. Importantly, if women are at risk of disordered eating and low GWG they would benefit from GWG guidance to prevent complications including small-for-gestational-age infants and seizure [16]. Two risk factors for gaining insufficient weight are a lack of provider advice about GWG, and provider advice to gain weight below that recommended in the guidelines [30]. Therefore, all women would benefit from weight gain advice, along with supplemented expert care for high risk groups, to make an informed choice regarding their health and GWG during pregnancy. More research is required to define the best way to approach GWG counselling for all women and ensure it is provided in a non-judgemental way and minimises stress for both parties.

Given the incomplete understandings of excessive GWG health impacts and concerns of ramifications of discussion it is therefore not surprising that only one-third of the midwives provided GWG guidelines while two-thirds did not, unless asked to do so by the woman. This, in part, may be explained by the elimination of regular weighing in antenatal practice removing a relevant cue to discuss weight. This is a concern given the evidence that suggests that the provision of guidelines appears to be successful in reducing excess GWG [27,30], meeting target GWG goals [30,49] and thus minimising complications [5,50]. Parallels and leads to translating guidelines into practice may be found in successful antenatal smoking cessation interventions [51]. In considering the health provider’s role in discussing smoking cessation, a common barrier was found to be personal beliefs that quitting smoking would have adverse effects on women’s psychological wellbeing [52] and that attempting to quit resulted in inordinate expenditure of emotional energy [53]. However, many studies have demonstrated that these concerns are unfounded [54-56]. It would be timely to assess the psychological impact of GWG interventions on pregnant women.

Clear evidence based guidelines regarding weighing and GWG are urgently required to clarify the clinical utility of maternal weight measurement and to allow for consistency of practice regarding the detection and management of excess GWG. Additionally, it is important that midwives are provided with education and support regarding the implementation of GWG guidelines. Lessons again may be taken from smoking cessation intervention clinical practice guidelines. These guidelines have been specifically designed for a public maternity care setting combined with an implementation program. Their implementation resulted in an increase in evidence-based practice with some indication of improved smoking behaviour for women [57].

Future interventions promoting healthy GWG may be enhanced by viewing the issue through a new lens. Up-skilling midwives regarding clinical importance and providing the tools to assess, promote and manage healthy GWG running parallel with healthy GWG interventions integrated with best-practice antenatal care were identified. Further research is required to ascertain pregnant women’s attitudes and beliefs regarding GWG, healthy eating and physical activity. Such research would ensure planned interventions are tailored to meet the needs of pregnant women during a period of life whereby they are likely to be motivated to optimise the health of her unborn child. While recent meta-analyses suggest that interventions based on physical activity and dietary counselling, often combined with weight monitoring, appeared to be successful in reducing GWG [27,28], however, the evidence remains weak [23-25]. Further study is required to ascertain effective, affordable and sustainable interventions for different risk groups and settings. Internet and texting via mobile phones are two examples of such interventions.

A strength of this study was the use of in-depth interviews. Face-to-face interviews enabled participants to discuss their views in a safe and private environment, offering and validating their experiences without having to explain or justify their views to co-workers. Previous overseas studies have utilised focus groups [27,34] with a mix of health providers or surveys [18,35], and these may feasibly prohibit full disclosure [42]. The regional nature of sampling allowed recruitment across many different midwifery practice areas covering a range of antenatal populations. Conversely, restricting the settings available for investigation may place some limits on the generalizability of the outcomes to wider settings. However, saturation of certain themes was obvious at an early stage in the study. The participants from different sites confirmed these themes allowing confidence in the conclusions. The regional nature of the sample must be considered when generalising to other regions and countries, however, the similarity of findings overseas [18,20,34] suggests that the issues discussed by the midwives are commonplace. The length of experience of the midwives, and the recruitment of many participants from a tertiary teaching hospital, may be both a strength and a weakness of this study. The study may have benefitted from the experience of the midwives, however, it may have also skewed the results towards those with more experience.

## Conclusion

This study found wide variation in midwives’ views and attitudes to GWG and excess GWG. While some midwives identified excess GWG as problematic and desired more information around the issue, the majority did not view it as an important clinical problem, despite being able to identify resultant co-morbidities associated with excess GWG. All midwives felt GWG held a low level of priority in the antenatal care agenda. These are unique data in the Australian context. In addition to improving the knowledge base, these data contribute to our understanding of the opportunities and challenges in promoting healthy GWG in the public health context. Importantly this study provides a foundation for further research into the experiences of GWG for women and health providers.

## Abbreviations

BMI: Body mass index; GP: General practitioner; GWG: Gestational weight gain; SMS: Short message service.

## Competing interests

The authors have no competing interests to report.

## Authors’ contributions

JW designed study, performed data collection, data analysis and drafted the manuscript. KC designed study, and contributed to data analysis and manuscript. SW contributed to data analysis and manuscript. PVP, DP and EH contributed to manuscript. All authors have critically reviewed the manuscript and approved the final manuscript.

## Pre-publication history

The pre-publication history for this paper can be accessed here:

http://www.biomedcentral.com/1471-2393/12/102/prepub

## Supplementary Material

Additional file 1 Interview questions. Click here for file
