# Supplementary material for: Excess gestational weight gain: an exploration of midwives’ views and practice
Source: BMC Pregnancy Childbirth. 2012 Sep 27;12:102. doi: 10.1186/1471-2393-12-102 (PMC3531303; doi:10.1186/1471-2393-12-102)
Supplement: Additional file 1 — Interview questions. [file 1471-2393-12-102-S1.pdf]

Additional file 1: Interview questions

Antenatal clinic director's administrative questions

1. Can you tell me how and when a pregnant woman would come to attend a midwife at the hospital? Prompts: number of visits; influence on number
2. Is there a schedule for what is covered at each visit? Prompts: If no, why not; history of schedule; how done and documented; measurements at visits; weight measurement and documentation; BMI calculation; guidance regarding appropriate amount of weight to gain.
3. What are the three biggest lifestyle related issues you see in your antenatal population?
4. How would women receive information and education about lifestyle issues and pregnancy at this hospital?
5. Does the department have policies or clinical guidelines specific to: Measurement of weight; GWG for all women; obesity in pregnancy; antenatal lifestyle education?

Midwives' questions

1. What comes to your mind when we talk about GWG?
2. On your first consultation with a pregnant women what is the focus of that visit? Prompt: weight measurement/body size/BMI
3. Is advice regarding healthy GWG offered during the course of the pregnancy? Prompt: Different pre-pregnancy BMI recommendations; guidelines used; reason for use/not use
4. Do women seek information about appropriate weight gain in their pregnancy?
5. Where do you get your information from about GWG?
6. Which lifestyle behaviours do you assess during the visits? Prompt: Types; process.
7. What are the triggers that alert you to excess GWG? Is it considered problematic?
8. What do you consider to be the most important implications of excess GWG?

9. In your opinion would excess GWG be identified? Prompt: Considered problematic; clinical pathway changes; differ for BMI?
10. What do you think influences your discussions with pregnant women around weight?
11. Who would you see as having the primary responsibility for education and intervention around GWG?
12. In a perfect scenario, how do you imagine you would be best supported to provide healthy lifestyle advice and support to pregnant women? Prompt: New technologies
13. What are the three key things you would want pregnant women to know about maintaining a healthy weight during pregnancy?
